# Systemic immune-inflammatory complex index as a novel predictor of sepsis prognosis: a retrospective cohort study using MIMIC-IV

**DOI:** 10.3389/fmed.2025.1608619

**Published:** 2025-06-30

**Authors:** Xueqing Wang, Yingxin Lin, Sheng Zhang, Junshi Wang, Bin Huang, Hua Luo, Lei Huang

**Affiliations:** Department of Intensive Care Unit (ICU), Peking University Shenzhen Hospital, Shenzhen, Guangdong, China

**Keywords:** systemic immune-inflammatory complex index, sepsis, mortality, scoring system, MIMIC-IV

## Abstract

**Purpose:**

Sepsis is a life-threatening condition with high mortality and morbidity, making its early detection is critical. Current diagnosis relies on the Sequential [sepsis-related] Organ Failure Assessment score, which is complex and time-consuming to determine. Herein, we proposed a novel index, the systemic immune-inflammatory complex index (SIICI), defined as (neutrophil × monocyte count) × 10^3^/(platelet × lymphocyte count), to predict illness severity, and we verified its prognostic value.

**Methods:**

All data were extracted from the Medical Information Mart for Intensive Care database IV (MIMIC-IV). Cox proportional hazards and Kaplan–Meier survival analyses were used to determine the association between target indices and 30- and 90-days mortality. Restricted cubic splines were used to reveal the linear relationship between indices and mortality. To assess the prognostic value of the SIICI, the systemic immune-inflammation index (SII) and systemic inflammation response index (SIRI), the area under the receiver operating characteristic curve (AUC), and the Youden index were measured and compared. Propensity score matching was used to reveal the association between the SIICI and secondary outcomes. Finally, subgroup analysis was performed to confirm the predictive ability of the SIICI.

**Results:**

We included 3,944 patients; among these, 609 (15.4%) and 663 (16.8%) patients had 30- and 90-days mortality, respectively. Our findings showed a strong association between the SIICI and mortality at 30 and 90 days in all models, which was more pronounced and better stratified than for the SIRI and SII. The *p*-value was < 0.05 in all cases; however, the SIICI was closer to a linear relationship with mortality than the SIRI or SII. Additionally, the SIICI had a higher AUC and Youden value than the other two indices. Moreover, a higher SIICI was positively associated with a longer stay in the intensive care unit or the hospital, an increased incidence of acute kidney injury, and greater use of renal replacement therapy and mechanical ventilation.

**Conclusion:**

The SIICI was positively associated with sepsis mortality and showed a better prognostic value than the SIRI and SII. The SIICI may be a promising complementary index to classical scoring systems for early assessment of patients with sepsis.

## 1 Introduction

Sepsis is a life-threatening organ dysfunction resulting from the host’s dysregulated systemic inflammatory response to infection. Sepsis accounts for nearly 10% of intensive care unit (ICU) admissions and represents a major health care problem and economic burden (1–3). Although the incidence and mortality of sepsis have declined in recent decades, the mortality rate remains high and is responsible for approximately one in five deaths worldwide (4). Owing to the severity and poor prognosis of sepsis and septic shock, early detection and intervention are critical. However, in the absence of a “gold standard” or single index for diagnosis (5), the Third International Consensus Definitions for Sepsis and Septic Shock (Sepsis-3) is still used to define sepsis as follows: infection and an increase in the Sequential [sepsis-related] Organ Failure Assessment (SOFA) score of 2 points or more (3). Although representative parameters of major systems are considered in the SOFA score, the advantages of simplicity, speed, and convenience are inevitably sacrificed. A similar scenario has been found in other recommended severity scoring systems, such as the Simplified Acute Physiology Score II (SAPS II).

Sepsis is a complex pathophysiologic process involving both pro- and anti-inflammatory responses, with activation of multiple cells and cytokines (2, 3, 5). Neutrophils and monocytes are activated successively and participate in a pro-inflammatory response to eliminate pathogens. An anti-inflammatory process is then initiated to limit tissue damage associated with apoptosis of T, B, and dendritic cells and impaired phagocytosis (2, 6). Studies have shown that lymphocytopenia is strongly associated with all-cause mortality (7–9). The imbalance between these pro- and anti-inflammatory responses is responsible for the severity of sepsis. Additionally, thrombocytopenia is common in critically ill patients, which is sepsis-induced in more than 50% of cases; furthermore, the degree of thrombocytopenia is one of the most important predictive indicators of sepsis mortality (10–14). Based on the above, recent studies have shown that the neutrophil-to-lymphocyte ratio (NLR), monocyte-to-lymphocyte ratio, and platelet-to-lymphocyte ratio (PLR) may be predictors of sepsis mortality; however, these reports are controversial (15–21). Derived from these indices, other novel composite indicators such as the systemic immune-inflammation index (SII; neutrophil × platelet/lymphocyte count) and systemic inflammation response index (SIRI; neutrophil × monocyte/lymphocyte count) have been shown to be more promising in predicting sepsis mortality (22–25).

Considering that neutrophils, lymphocytes, monocytes, and platelets are the most representative components involved in the inflammatory process of sepsis, we aimed to propose a more comprehensive index that includes all these factors. Moreover, because the reduction of lymphocytes and platelets is intimately related to sepsis severity, it is theoretically reasonable to name the new indicator the systemic immune-inflammatory complex index (SIICI), calculated as neutrophil count × monocyte count × 10^3^/(platelet count × lymphocyte count). The aim of this study was to demonstrate the prognostic value of the SIICI for sepsis mortality and compare it with those of the SIRI and SII.

## 2 Materials and methods

### 2.1 Data source

All data in this retrospective cohort study were extracted from the Medical Information Mart for Intensive Care database IV (MIMIC-IV) version 2.2, which includes more than 73,181 ICU admissions at Beth Israel Deaconess Medical Center (BIDMC; Boston, MA, United States) from 2008 to 2019. The Collaborative Institutional Training Initiative license was previously obtained to access the database (ID: 13285556), and the use of the database was approved by the institutional review boards of the Massachusetts Institute of Technology and BIDMC. All procedures involving human participants conformed to the ethical standards of the institutional and national research committee, as well as the Helsinki Declaration in 1964 and its subsequent amendments or comparable ethical standards. The requirement for informed consent was waived owing to the de-identification of all patient data.

### 2.2 Population and exclusion criteria

**FIGURE 1:**
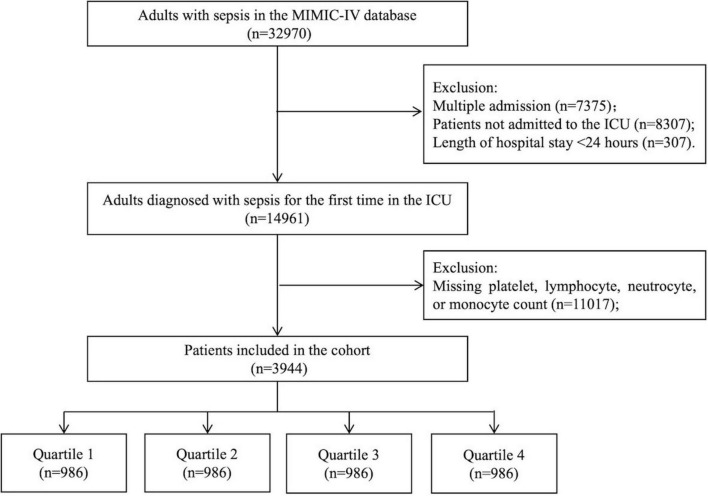
Flow chart.

Patients with a first diagnosis of sepsis who were admitted to the ICU were included in this study. Sepsis was defined according to Sepsis 3.0, which is included in a special file of the MIMIC-IV database. The exclusion criteria were as follows: (1) multiple admissions; (2) patients not admitted to the ICU; (3) hospital stay less than 24 h; and (3) missing data for platelet, lymphocyte, neutrophil, or monocyte count within the first 24 h after admission. Finally, 3944 patients were included and divided into four groups according to quartiles of the log2-transformed SIICI, SIRI, or SII. The screening process is shown in Figure 1.

### 2.3 Data collection

All data were acquired from MIMIC-IV using Structured Query Language (SQL) with PostgreSQL version 16.2. The information included (1) length of hospital and ICU stay; (2) patient demographics: age, sex, race and ethnicity, and marital status; (3) vital signs: heart rate, systolic blood pressure, diastolic blood pressure, mean arterial pressure (MAP), respiratory rate, and oxygen saturation; (4) laboratory indicators: lymphocyte count, monocyte count, neutrophil count, platelet count, white blood cell count, hematocrit, hemoglobin, albumin, serum creatinine, urea nitrogen, total bilirubin, glucose, international normalized ratio, activated partial thromboplastin time, sodium, potassium, bicarbonate, lactate, and partial pressure of oxygen; (5) comorbidities: acute kidney injury (AKI), chronic kidney disease (CKD), chronic obstructive pulmonary disease, respiratory failure, ischemic heart disease (IHD), heart failure, shock, multiple organ dysfunction syndrome, connective tissue disease (CTD), cirrhosis, thrombocytopenia (TCP), hematological tumors (HTs), metastatic carcinoma (MC), and AIDS; (6) operations: ventilation and renal replacement therapy (RRT); and (7) scoring systems: SOFA and SAPS II. Vital signs, laboratory indicators, and scoring system information were collected within the first 24 hours after admission. All comorbidities and operations were defined according to the International Classification of Diseases, Ninth and Tenth revision codes (ICD-9 and ICD-10, respectively).

Multiple imputation based on the random forest model (*missForest*) was used with less than 20% missing data; variables were excluded with more than 50% missing data. Indices with between 20 and 50% missing data could be converted to categorical variables and included as dummy variables, which were not included in this study.

The related parameters were calculated using the following formulas (in the first formula, the constant of 10^3^ was introduced to scale the data to increase the ease of calculation and the readability of numerical values):


SIICI=(neutrophil×monocytecount)×10/3

(platelet×lymphocytecount)

SIRI=(neutrophil×monocytecount)/lymphocytecount

SII=(neutrophil×plateletcount)/lymphocytecount

### 2.4 Outcomes

Primary outcomes were 30- and 90-days mortality. Secondary outcomes were length of ICU and hospital stay, incidence of shock and AKI, and use of RRT and mechanical ventilation. AKI was defined according to the Kidney Disease: Improving Global Outcomes guidelines.

### 2.5 Statistical analysis

The Shapiro–Wilk test was used to assess normality. All continuous variables in this study followed a non-normal distribution, which was described using median and interquartile range (IQR) and compared using the Mann–Whitney U-test or Kruskal–Wallis test. Categorical variables were described using frequency and percentage and compared using the Pearson chi-square test or Fisher’s exact test.

Cox proportional hazards models were applied to calculate the hazard ratio (HR) and 95% confidence interval (CI) to determine and compare the association between the SIICI, SIRI, and SII indices and 30- or 90-days mortality. Confounders were included and adjusted in multiple models. Model 1 was unadjusted. Model 2 was adjusted for age, sex, marital status, and race and ethnicity. Model 3 was adjusted for age, sex, marital status, race and ethnicity, heart rate, systolic blood pressure, diastolic blood pressure, MAP, respiratory rate, hematocrit, hemoglobin, albumin, MC, IHD, CKD, and cirrhosis.

A restricted cubic spline model was used to determine the linear or non-linear relationship between the SIICI, SIRI, or SII and the primary outcomes. A Kaplan–Meier survival analysis curve was plotted to express the association between different quartiles of novel indices and sepsis mortality, with *p-*values calculated using the log-rank test.

To further assess the prognostic value of the SIICI, SIRI, and SII, the receiver operating characteristic (ROC) curve was plotted, and the area under the ROC curve (AUC) was determined. Additionally, the sensitivity, specificity, and Youden index (calculated as: sensitivity + specificity − 1) were determined.

The secondary endpoints were evaluated based on propensity score matching (PSM), which was performed according to Model 3. Subgroup analyses were performed to assess the impact and consistency of the SIICI in different groups of patients with sepsis.

All statistical analyses were conducted using R software version 4.3.3 (The R Project for Statistical Computing, Vienna, Austria). We considered *p* < 0.05 statistically significant.

## 3 Results

### 3.1 Baseline characteristics

A total of 3,944 patients were included in this study, and 59% of the population was male. The median age was 66 years, 59% of patients were white race and ethnicity, and 40% were married. The 30 and 90-days mortality was 15.4 and 16.8%, respectively. The length of ICU and hospital stay was 4 and 11 days, respectively. The median [IQR] SIICI (log) index was 4.96 [3.80, 6.19], and the quartiles were as follows: Q1, 0.00–3.80; Q2, 3.80–4.96; Q3, 4.96–6.19; and Q4, 6.19–13.23. The median [IQR] SIRI (log) index was 2.69 [1.69, 3.75], and the quartiles were as follows: Q1, 0.00–1.69; Q2, 1.69–2.69; Q3, 2.69–3.75; and Q4, 3.75–8.51. The median [IQR] SII (log) index was 10.37 [9.42, 11.46], and the quartiles were as follows: Q1, 0.07–9.42; Q2, 9.42–10.37; Q3, 10.37–11.46; and Q4, 11.46–15.51.

When comparing general characteristics between survivors and non-survivors at 30 and 90 days, non-survivors tended to have longer ICU and hospital stays; older age; worse vital signs; higher levels of the SIICI, SIRI, and SII; increased incidence of multiorgan dysfunction; and more use of ventilation and RRT. Not surprisingly, non-survivors had a more severe condition, as assessed using the SOFA score and SAPS II. The only difference between 30- and 90-days mortality was that enrolled patients with HTs showed a significant increase in 90-day mortality but not in 30-day mortality (Table 1).

**TABLE 1:** Baseline characteristics of patients with sepsis according to primary outcomes.

Characteristics	Overall, *n* = 3,944^1^	30–day mortality			90–day mortality		
		**Survivors,** ***n* = 3,335^1^**	**Non–survivors,** ***n* = 609^1^**	***p*–value**	**Survivors,** ***n* = 3,281^1^**	**Non–survivors,** ***n* = 663^1^**	***p*–value**
ICU stay, days	4 [2, 8]	3 [2, 8]	5 [3, 10]	<0.001	3 [2, 8]	5 [3, 11]	<0.001
Hospital stay, days	11 [6, 21]	12 [7, 22]	9 [4, 15]	<0.001	12 [7, 21]	10 [4, 18]	<0.001
Marital status				<0.001			<0.001
Married	1,588 (40%)	1,392 (42%)	196 (32%)		1,373 (42%)	215 (32%)	
Not married	2,356 (60%)	1,943 (58%)	413 (68%)		1,908 (58%)	448 (68%)	
Race and ethnicity				<0.001			<0.001
Non-white	1,621 (41%)	1,290 (39%)	331 (54%)		1,262 (38%)	359 (54%)	
White	2,323 (59%)	2,045 (61%)	278 (46%)		2,019 (62%)	304 (46%)	
Sex				0.7			0.4
Female	1,600 (41%)	1,349 (40%)	251 (41%)		1,321 (40%)	279 (42%)	
Male	2,344 (59%)	1,986 (60%)	358 (59%)		1,960 (60%)	384 (58%)	
Age, years	66 [55, 76]	66 [54, 76]	68 [56, 79]	<0.001	66 [54, 76]	68 [56, 79]	0.001
**Vital Signs**							
Heart rate, beats/min	87 [77, 102]	86 [76, 101]	95 [80, 110]	<0.001	86 [76, 101]	95 [81, 110]	<0.001
Systolic blood pressure, mmHg	117 [103, 134]	117 [103, 134]	118 [103, 134]	0.8	117 [103, 134]	118 [102, 135]	0.8
Diastolic blood pressure, mmHg	67 [57, 79]	67 [57, 78]	70 [58, 84]	<0.001	67 [57, 79]	70 [58, 84]	0.002
MAP, mmHg	80 [69, 93]	79 [69, 92]	82 [71, 96]	0.001	79 [69, 92]	82 [70, 96]	0.003
Respiratory rate, times/min	19 [15, 23]	18 [15, 23]	21 [17, 26]	<0.001	18 [15, 23]	21 [17, 26]	<0.001
SaO_2_,%	98.0 [95.0, 100.0]	98.0 [95.0, 100.0]	97.0 [94.0, 100.0]	<0.001	98.0 [95.0, 100.0]	97.0 [94.0, 100.0]	<0.001
**Laboratory indicators**							
SIICI (log)	4.96 [3.80, 6.19]	4.84 [3.71, 5.98]	5.81 [4.55, 7.05]	<0.001	4.82 [3.69, 5.96]	5.76 [4.55, 6.99]	<0.001
SIRI (log)	2.69 [1.69, 3.75]	2.59 [1.63, 3.62]	3.44 [2.18, 4.42]	<0.001	2.56 [1.63, 3.60]	3.42 [2.20, 4.40]	<0.001
**Laboratory indicators**							
SII (log)	10.37 [9.42, 11.46]	10.31 [9.38, 11.34]	10.88 [9.68, 11.96]	<0.001	10.30 [9.38, 11.32]	10.86 [9.71, 11.95]	<0.001
Lymphocyte count, 10^9^/L	1.21 [0.73, 1.85]	1.25 [0.77, 1.91]	0.93 [0.51, 1.45]	<0.001	1.25 [0.78, 1.92]	0.94 [0.52, 1.47]	<0.001
Monocyte count, 10^9^/L	0.71 [0.42, 1.08]	0.69 [0.41, 1.06]	0.80 [0.43, 1.23]	<0.001	0.69 [0.41, 1.06]	0.80 [0.44, 1.23]	<0.001
Neutrocyte count, 10^9^/L	10 [6, 14]	9 [6, 14]	11 [7, 16]	<0.001	9 [6, 14]	11 [7, 16]	<0.001
Platelet count, 10^9^/L	181 [129, 238]	183 [132, 239]	163 [101, 235]	<0.001	183[132, 238]	165 [105, 236]	<0.001
White blood cell count, 10^9^/L	11 [8, 16]	11 [8, 16]	13 [9, 19]	<0.001	11 [8, 16]	13 [9, 18]	<0.001
Hematocrit,%	34 [29, 39]	34 [29, 39]	32 [27, 38]	<0.001	34 [29, 39]	32 [27, 38]	<0.001
Hemoglobin, g/Dl	11.00 [9.30, 12.80]	11.10 [9.40, 12.80]	10.40 [8.70, 12.30]	<0.001	11.10 [9.40, 12.80]	10.50 [8.70, 12.30]	<0.001
Albumin, g/dL	3.10 [2.70, 3.57]	3.20 [2.80, 3.60]	2.90 [2.50, 3.31]	<0.001	3.20 [2.80, 3.60]	2.90 [2.50, 3.33]	<0.001
Serum creatinine, mg/dL	1.00 [0.80, 1.50]	1.00 [0.70, 1.40]	1.30 [0.90, 2.10]	<0.001	1.00 [0.70, 1.40]	1.30 [0.90, 2.10]	<0.001
Urea nitrogen, mg/dL	19 [14, 31]	19 [14, 29]	27 [17, 46]	<0.001	19 [14, 28]	27 [17, 45]	<0.001
Total bilirubin, mg/dL	0.70 [0.40, 1.30]	0.70 [0.40, 1.20]	0.90 [0.50, 2.10]	<0.001	0.70 [0.40, 1.18]	0.90 [0.50, 2.10]	<0.001
Glucose, mg/dL	125 [102, 162]	123 [101, 157]	139 [106, 190]	<0.001	123[101, 157]	137 [106, 187]	<0.001
INR	1.30 [1.10, 1.50]	1.20 [1.10, 1.48]	1.40 [1.20, 1.90]	<0.001	1.20 [1.10, 1.42]	1.40 [1.20, 1.90]	<0.001
APTT, s	31 [27, 37]	30 [27, 36]	33 [28, 43]	<0.001	30 [27, 36]	32 [28, 42]	<0.001
Sodium, mmol/L	139.0 [136.0, 142.0]	139.0 [136.0, 141.0]	138.0 [135.0, 142.0]	0.047	139.0 [136.0, 141.0]	138.0 [134.0, 142.0]	0.014
Potassium, mmol/L	4.10 [3.70, 4.60]	4.10 [3.80, 4.50]	4.30 [3.70, 4.80]	0.003	4.10 [3.80, 4.50]	4.30 [3.70, 4.80]	0.003
Bicarbonate, mmol/L	22.0 [20.0, 25.0]	23.0 [20.0, 25.0]	21.0 [18.0, 24.0]	<0.001	23.0 [20.0, 25.0]	21.0 [18.0, 24.0]	<0.001
Lactate, mmol/L	1.70 [1.20, 2.50]	1.60 [1.20, 2.30]	2.30 [1.60, 4.30]	<0.001	1.60 [1.20, 2.30]	2.30 [1.60, 4.20]	<0.001
PO_2_, mmHg	104 [54, 224]	112 [58, 254]	78 [48, 128]	<0.001	112[58, 256]	78 [48, 130]	<0.001
**Comorbidities**							
AKI	1,779 (45%)	1,332 (40%)	447 (73%)	<0.001	1,291 (39%)	488 (74%)	<0.001
CKD	772 (20%)	612 (18%)	160 (26%)	<0.001	602(18%)	170 (26%)	<0.001
COPD	530 (13%)	417 (13%)	113 (19%)	<0.001	410(12%)	120 (18%)	<0.001
Respiratory failure, RF	1,859 (47%)	1,395 (42%)	464 (76%)	<0.001	1,354 (41%)	505 (76%)	<0.001
Ischemic heart disease, IHD	680 (17%)	530 (16%)	150 (25%)	<0.001	521(16%)	159 (24%)	<0.001
Heart failure, HF	1,100 (28%)	877 (26%)	223 (37%)	<0.001	863(26%)	237 (36%)	<0.001
Shock	632 (16%)	405 (12%)	227 (37%)	<0.001	388(12%)	244 (37%)	<0.001
Connective tissue disease, CTD	85 (2.2%)	68 (2.0%)	17 (2.8%)	0.2	65 (2.0%)	20 (3.0%)	0.094
Cirrhosis	9 (0.2%)	5 (0.1%)	4 (0.7%)	0.037	5 (0.2%)	4 (0.6%)	0.049
Thrombocytopenia, TCP	967 (25%)	770 (23%)	197 (32%)	<0.001	752(23%)	215 (32%)	<0.001
Hematologic tumor, HT	126 (3.2%)	99 (3.0%)	27 (4.4%)	0.059	89 (2.7%)	37 (5.6%)	<0.001
Metastatic carcinoma, MC	152 (3.9%)	104 (3.1%)	48 (7.9%)	<0.001	100(3.0%)	52 (7.8%)	<0.001
AIDS	17 (0.4%)	16 (0.5%)	1 (0.2%)	0.5	15 (0.5%)	2 (0.3%)	0.8
**Operations**							
Ventilation	2,262 (57%)	1,749 (52%)	513 (84%)	<0.001	1,704 (52%)	558 (84%)	<0.001
RRT	499 (13%)	288 (8.6%)	211 (35%)	<0.001	269(8.2%)	230 (35%)	<0.001
**Scoring systems**							
SOFA	4 [2, 7]	4 [2, 6]	8 [5, 11]	<0.001	4 [2, 6]	8 [5, 11]	<0.001
SAPS II	40 [33, 47]	38 [32, 45]	47 [41, 54]	<0.001	38 [32, 45]	47 [41, 54]	<0.001

MAP, Mean arterial pressure; SaO_2_, Oxygen saturation; INR, International normalized ratio; APTT, Activated partial thromboplastin time; PO_2_, Partial pressure of oxygen; AKI, Acute kidney injury; CKD, Chronic kidney disease; COPD, Chronic obstructive pulmonary disease; AIDS, Acquired immunodeficiency syndrome; RRT, Renal replacement therapy; SOFA, Sequential organ failure assessment; SAPS II, Simplified acute physiology score II.

^1^Continuous variables are described as the median and interquartile range (IQR) (median [IQR]), categorical variables are described as frequencies and percentages [n (%)].

To observe the baseline characteristics with different levels of the SIICI, SIRI, and SII and to horizontally compare the difference among the above indices, the indices were stratified into four quartiles of 986 patients each. All indices showed a positive association with increased mortality, longer ICU and hospital stay, worse vital signs, and higher SOFA and SAPS II scores. A similar trend remained for laboratory parameters, except that hematocrit and hemoglobin showed no association with SIRI levels, and potassium showed a negative correlation with the stratified SIICI. In terms of comorbidities, IHD and MC showed a strong relationship with the SII, which was not observed in the other indices. TCP had no relationship with the SIRI, which was the opposite of the others. Higher levels of the SIICI, SIRI, or SII tended to be associated with an increased risk of shock and increased incidence of renal, respiratory, and cardiac failure, as well as greater use of ventilation and RRT; however, no difference was found in CTD and HT (Table 2; Supplementary Tables S1, S2).

**TABLE 2:** Baseline characteristics of patients with sepsis according to the quartiles of the SIICI (log).

Characteristics	Overall, *n* = 3,944^1^	Q1, *n* = 986^1^	Q2, *n* = 986^1^	Q3, *n* = 986^1^	Q4, *n* = 986^1^	*p*–value
30–day mortality	609 (15%)	88 (8.9%)	110 (11%)	152 (15%)	259 (26%)	<0.001
90–day mortality	663 (17%)	97 (9.8%)	122 (12%)	165 (17%)	279 (28%)	<0.001
In–hospital mortality	665 (17%)	97 (9.8%)	122 (12%)	167 (17%)	279 (28%)	<0.001
ICU stay, day	4 [2,8]	3 [1,6]	3 [2,8]	4 [2,9]	5 [2,9]	<0.001
Hospital stay, day	11 [6,21]	9 [6,18]	11 [6,20]	12 [7,21]	13 [7,23]	<0.001
Marital status						<0.001
Married	1,588 (40%)	424 (43%)	440 (45%)	374 (38%)	350 (35%)	
Not married	2,356 (60%)	562 (57%)	546 (55%)	612 (62%)	636 (65%)	
Race and ethnicity						0.3
Non-white	1,621 (41%)	392 (40%)	402 (41%)	396 (40%)	431 (44%)	
White	2,323 (59%)	594 (60%)	584 (59%)	590 (60%)	555 (56%)	
Sex						0.068
Female	1,600 (41%)	434 (44%)	396 (40%)	379 (38%)	391 (40%)	
Male	2,344 (59%)	552 (56%)	590 (60%)	607 (62%)	595 (60%)	
Age, years	66 [55, 76]	67 [55, 75]	66 [55, 75]	67 [55, 77]	66 [54, 77]	0.6
**Vital signs**						
Heart rate, beats/min	87 [77, 102]	82 [75, 96]	85 [76, 100]	89 [78, 105]	94 [79, 108]	<0.001
Systolic blood pressure, mmHg	117 [103, 134]	117 [101, 131]	116 [101, 131]	118 [104, 136]	119 [104, 136]	0.004
Diastolic blood pressure, mmHg	67 [57, 79]	66 [55, 77]	66 [57, 78]	69 [58, 81]	67 [58, 80]	<0.001
MAP, mmHg	80 [69, 93]	78 [68, 92]	78 [69, 91]	82 [71, 95]	81 [70, 93]	<0.001
Respiratory rate, times/min	19 [15, 23]	17 [14, 22]	18 [15, 23]	19 [16, 23]	20 [17, 25]	<0.001
SaO_2_,%	98.0 [95.0, 100.0]	99.0 [96.0, 100.0]	99.0 [96.0, 100.0]	98.0 [95.0, 100.0]	97.0 [94.0, 100.0]	<0.001
**Laboratory indicators**						
SIICI	30 [13, 72]	7 [4, 10]	21 [16, 25]	45 [37, 56]	135 [99, 228]	<0.001
SIICI (log)	4.96 [3.80, 6.19]	3.00 [2.35, 3.42]	4.44 [4.10, 4.70]	5.51 [5.26, 5.83]	7.09 [6.64, 7.84]	<0.001
Lymphocyte count, 10^9^/L	1.21 [0.73, 1.85]	1.66 [1.07, 2.44]	1.44 [0.96, 2.02]	1.10 [0.75, 1.57]	0.75 [0.45, 1.20]	<0.001
Monocyte count, 10^9^/L	0.71 [0.42, 1.08]	0.36 [0.20, 0.57]	0.64 [0.44, 0.90]	0.86 [0.59, 1.16]	1.13 [0.78, 1.65]	<0.001
Neutrocyte count, 10^9^/L	10 [6, 14]	6 [4, 9]	9 [6, 12]	11 [8, 14]	15 [11, 20]	<0.001
Platelet count, 10^9^/L	181 [129, 238]	193 [146, 256]	192 [137, 245]	185 [134, 236]	150 [96, 207]	<0.001
White blood cell count, 10^9^/L	11 [8, 16]	8 [6, 11]	11 [8, 14]	12 [9, 16]	16 [11, 22]	<0.001
Hematocrit,%	34 [29, 39]	35 [29, 39]	34 [29, 39]	34 [29, 39]	33 [28, 38]	0.002
Hemoglobin, g/dL	11.00 [9.30, 12.80]	11.30 [9.40, 12.90]	11.10 [9.23, 12.80]	11.00 [9.30, 12.80]	10.60 [9.00, 12.50]	0.001
Albumin, g/dL	3.10 [2.70, 3.57]	3.30 [2.90, 3.88]	3.20 [2.80, 3.60]	3.10 [2.70, 3.50]	2.90 [2.60, 3.30]	<0.001
Serum creatinine, mg/dL	1.00 [0.80, 1.50]	0.90 [0.70, 1.20]	1.00 [0.70, 1.30]	1.00 [0.80, 1.50]	1.20 [0.80, 2.00]	<0.001
Urea nitrogen, mg/dL	19 [14, 31]	18 [13, 25]	18 [13, 28]	20 [14, 32]	25 [16, 41]	<0.001
Total bilirubin, mg/dL	0.70 [0.40, 1.30]	0.60 [0.40, 0.90]	0.66 [0.40, 1.09]	0.70 [0.48, 1.30]	1.00 [0.50, 2.30]	<0.001
Glucose, mg/dL	125 [102, 162]	117 [98, 151]	122 [102, 152]	129 [106, 170]	133 [106, 173]	<0.001
INR	1.30 [1.10, 1.50]	1.20 [1.10, 1.40]	1.20 [1.10, 1.50]	1.30 [1.10, 1.50]	1.40 [1.20, 1.79]	<0.001
APTT, s	31 [27, 37]	31 [28, 37]	30 [27, 36]	30 [27, 37]	31 [27, 38]	0.042
Sodium, mmol/L	139.0 [136.0, 142.0]	139.0 [137.0, 142.0]	139.0 [136.0, 141.0]	139.0 [136.0, 142.0]	138.0 [135.0, 141.0]	<0.001
Potassium, mmol/L	4.10 [3.70, 4.60]	4.10 [3.80, 4.40]	4.10 [3.80, 4.50]	4.10 [3.70, 4.60]	4.20 [3.70, 4.70]	0.12
Bicarbonate, mmol/L	22.0 [20.0, 25.0]	23.0 [21.0, 25.0]	23.0 [20.0, 25.0]	22.0 [19.0, 25.0]	21.0 [18.0, 24.0]	<0.001
Lactate, mmol/L	1.70 [1.20, 2.50]	1.50 [1.10, 2.10]	1.60 [1.20, 2.20]	1.80 [1.30, 2.60]	2.10 [1.50, 3.20]	<0.001
PO_2_, mmHg	104 [54, 224]	180 [78, 351]	118 [61, 285]	94 [49, 170]	77 [46, 127]	<0.001
**Comorbidities**						
AKI	1,779 (45%)	322 (33%)	388 (39%)	460 (47%)	609 (62%)	<0.001
CKD	772 (20%)	146 (15%)	198 (20%)	195 (20%)	233 (24%)	<0.001
COPD	530 (13%)	97 (9.8%)	132 (13%)	146 (15%)	155 (16%)	<0.001
Respiratory failure, RF	1,859 (47%)	327 (33%)	424 (43%)	510 (52%)	598 (61%)	<0.001
Ischemic heart disease, IHD	680 (17%)	164 (17%)	162 (16%)	190 (19%)	164 (17%)	0.3
Heart failure, HF	1,100 (28%)	232 (24%)	263 (27%)	301 (31%)	304 (31%)	<0.001
Shock	632 (16%)	107 (11%)	134 (14%)	168 (17%)	223 (23%)	<0.001
Connective tissue disease, CTD	85 (2.2%)	22 (2.2%)	20 (2.0%)	15 (1.5%)	28 (2.8%)	0.2
Cirrhosis	9 (0.2%)	2 (0.2%)	2 (0.2%)	3 (0.3%)	2 (0.2%)	> 0.9
Thrombocytopenia, TCP	967 (25%)	188 (19%)	208 (21%)	221 (22%)	350 (35%)	<0.001
Hematologic tumor, HT	126 (3.2%)	43 (4.4%)	22 (2.2%)	21 (2.1%)	40 (4.1%)	0.004
Metastatic carcinoma, MC	152 (3.9%)	36 (3.7%)	37 (3.8%)	34 (3.4%)	45 (4.6%)	0.6
AIDS	17 (0.4%)	14 (1.4%)	0 (0%)	2 (0.2%)	1 (0.1%)	<0.001
**Operations**						
Ventilation	2,262 (57%)	493 (50%)	548 (56%)	597 (61%)	624 (63%)	<0.001
RRT	499 (13%)	77 (7.8%)	92 (9.3%)	120 (12%)	210 (21%)	<0.001
**Scoring systems**						
SOFA	4 [2, 7]	4 [2, 6]	4 [2, 6]	4 [2, 6]	5 [3, 8]	<0.001
SAPS II	40 [33, 47]	36 [31, 42]	38 [32, 45]	41 [34, 47]	44 [38, 51]	<0.001

SIICI (log) quartiles: Q1, 0.00–3.80; Q2, 3.80–4.96; Q3, 4.96–6.19; Q4, 6.19–13.23. MAP, Mean arterial pressure; SaO_2_, Oxygen saturation; INR, International normalized ratio; APTT, Activated partial thromboplastin time; PO_2_, Partial pressure of oxygen; AKI, Acute kidney injury; CKD, Chronic kidney disease; COPD, Chronic obstructive pulmonary disease; AIDS, Acquired immunodeficiency syndrome; RRT, Renal replacement therapy; SOFA, Sequential organ failure assessment; SAPS II, Simplified acute physiology score II.

^1^Continuous variables are described as the median and interquartile range (IQR) (median [IQR]), categorical variables are described as frequencies and percentages [n (%)].

### 3.2 Association between the SIICI, SIRI, or SII and sepsis mortality

Cox proportional hazards models unveiled a significant association between the SIICI and 30 or 90-days mortality in all models (30-day mortality: Model 1, HR [95% CI], 1.192 [1.143, 1.243]; Model 2, 1.186 [1.137, 1.236]; and Model 3, 1.162 [1.114, 1.212], *p* < 0.001 in all models; 90-day mortality: Model 1, HR [95% CI], 1.175 [1.129, 1.223]; Model 2, 1.171 [1.125, 1.219]; and Model 3, 1.150 [1.104, 1.197], *p* < 0.001 in all models). A similar trend was observed in the SIRI (30-day mortality: Model 1, HR [95% CI], 1.203 [1.141, 1.268]; Model 2, 1.189 [1.128, 1.254]; and Model 3, 1.153 [1.093, 1.216], *p* < 0.001 in all models; 90-day mortality: Model 1, HR [95% CI], 1.191 [1.133, 1.253]; Model 2, 1.181 [1.123, 1.242]; and Model 3, 1.149 [1.092, 1.209], *p* < 0.001 in all models). Although the SII also showed a difference in all models, the HR in each one was markedly lower than the SIICI or SIRI, indicating a smaller effect on mortality prognosis (30-day mortality: Model 1, HR [95% CI], 1.100 [1.049, 1.154], *p* < 0.001; Model 2, 1.086 [1.036, 1.140], *p* < 0.001; and Model 3, 1.051 [1.004, 1.100], *p* = 0.032; 90-day mortality: Model 1, HR [95% CI], 1.092 [1.044, 1.142], *p* < 0.001; Model 2, 1.079 [1.031, 1.129], *p* < 0.001; and Model 3, 1.049 [1.004, 1.095], *p* = 0.032; Table 3).

**TABLE 3:** Cox proportional hazards analysis for SIICI, SIRI, SII, and sepsis mortality.

Primary outcome	Model 1		Model 2		Model 3	
	**HR [95% CI]** ^1^	***p*–value**	**HR [95% CI]** ^1^	***p*–value**	**HR [95% CI]** ^1^	***p*–value**
**SIICI (log)**						
30–day mortality continuous variable per 1 unit	1.192 [1.143, 1.243]	<0.001	1.186 [1.137, 1.236]	<0.001	1.162 [1.114, 1.212]	<0.001
**Quartiles^a^**						
Q1 (*n* = 986)	–		–		–	
Q2 (*n* = 986)	1.153 [0.871, 1.526]	0.3	1.138 [0.860, 1.507]	0.4	1.117 [0.843, 1.481]	0.4
Q3 (*n* = 986)	1.497 [1.151, 1.947]	0.003	1.482 [1.139, 1.929]	0.003	1.415 [1.085, 1.845]	0.01
Q4 (*n* = 986)	2.430 [1.907, 3.096]	<0.001	2.403 [1.886, 3.062]	<0.001	2.159 [1.688, 2.760]	<0.001
P for trend		<0.001		< 0.001		<0.001
90–day mortality continuous variable per 1 unit	1.175 [1.129, 1.223]	<0.001	1.171 [1.125, 1.219]	<0.001	1.150 [1.104, 1.197]	<0.001
**Quartiles^a^**						
Q1 (*n* = 986)	–		–		–	
Q2 (*n* = 986)	1.176 [0.901, 1.535]	0.2	1.164 [0.891, 1.520]	0.3	1.132 [0.866, 1.480]	0.4
Q3 (*n* = 986)	1.474 [1.147, 1.894]	0.002	1.466 [1.140, 1.885]	0.003	1.411 [1.095, 1.819]	0.008
Q4 (*n* = 986)	2.352 [1.866, 2.964]	<0.001	2.350 [1.864, 2.963]	<0.001	2.127 [1.682, 2.689]	<0.001
P for trend		<0.001		< 0.001		<0.001
**SIRI (log)**						
30–day mortality continuous variable per 1 unit	1.203 [1.141, 1.268]	<0.001	1.189 [1.128, 1.254]	<0.001	1.153 [1.093, 1.216]	<0.001
**Quartiles^b^**						
Q1 (*n* = 986)	–		–		–	
Q2 (*n* = 986)	0.990 [0.754, 1.300]	> 0.9	0.965 [0.734, 1.268]	0.8	0.942 [0.716, 1.240]	0.7
Q3 (*n* = 986)	1.268 [0.986, 1.632]	0.065	1.246 [0.968, 1.604]	0.088	1.166 [0.904, 1.503]	0.2
Q4 (n = 986)	2.008 [1.589, 2.537]	<0.001	1.915 [1.515, 2.421]	<0.001	1.689 [1.332, 2.140]	<0.001
P for trend		<0.001		< 0.001		<0.001
90–day mortality continuous variable per 1 unit	1.191 [1.133, 1.253]	<0.001	1.181 [1.123, 1.242]	<0.001	1.149 [1.092, 1.209]	<0.001
**Quartiles^b^**						
Q1 (*n* = 986)	–		–		–	
Q2 (*n* = 986)	0.973 [0.748, 1.266]	0.8	0.951 [0.731, 1.238]	0.7	0.915 [0.702, 1.193]	0.5
Q3 (*n* = 986)	1.316 [1.036, 1.671]	0.024	1.304 [1.026, 1.657]	0.03	1.224 [0.961, 1.558]	0.1
Q4 (*n* = 986)	1.977 [1.581, 2.473]	<0.001	1.909 [1.526, 2.389]	<0.001	1.702 [1.357, 2.136]	<0.001
P for trend		<0.001		< 0.001		<0.001
**SIICI (log)**						
**SII (log)**						
**30–day mortality continuous variable per 1 unit**	1.100 [1.049, 1.154]	<0.001	1.086 [1.036, 1.140]	<0.001	1.051 [1.004, 1.100]	0.032
**Quartiles^c^**						
Q1 (*n* = 986)	–		–		–	
Q2 (*n* = 986)	0.829 [0.641, 1.072]	0.2	0.818 [0.632, 1.058]	0.13	0.851 [0.657, 1.104]	0.2
Q3 (*n* = 986)	1.025 [0.808, 1.300]	0.8	0.999 [0.787, 1.267]	> 0.9	0.955 [0.751, 1.214]	0.7
Q4 (*n* = 986)	1.604 [1.288, 1.998]	<0.001	1.509 [1.210, 1.881]	<0.001	1.318 [1.052, 1.652]	0.017
P for trend		<0.001		< 0.001		0.011
**90–day mortality continuous variable per 1 unit**	1.092 [1.044, 1.142]	<0.001	1.079 [1.031, 1.129]	<0.001	1.049 [1.004, 1.095]	0.03
**Quartiles^c^**						
Q1 (*n* = 986)	–		–		–	
Q2 (*n* = 986)	0.861 [0.673, 1.100]	0.2	0.846 [0.662, 1.083]	0.2	0.884 [0.689, 1.133]	0.3
Q3 (*n* = 986)	1.059 [0.844, 1.330]	0.6	1.035 [0.824, 1.300]	0.8	1.003 [0.796, 1.264]	> 0.9
Q4(*n* = 986)	1.639 [1.327, 2.025]	<0.001	1.543 [1.248, 1.909]	<0.001	1.367 [1.099, 1.699]	0.005
P for trend		<0.001		< 0.001		0.003

Model 1: Unadjusted. Model 2: Adjusted for age, sex, race and ethnicity, and marital status. Model 3: Adjusted for age, sex, race and ethnicity, marital status, heart rate, systolic blood pressure, diastolic blood pressure, mean arterial pressure, respiratory rate, hematocrit, hemoglobin, albumin, metastatic carcinoma, ischemic heart disease, chronic kidney disease, and cirrhosis.

^1^HR, Hazard ratio; CI, Confidence interval.

*^a^*SIICI (log) quartiles: Q1, 0.00–3.80; Q2, 3.80–4.96; Q3, 4.96–6.19; Q4, 6.19–13.23.

*^b^*SIRI (log) quartiles: Q1, 0.00–1.69; Q2, 1.69–2.69; Q3, 2.69–3.75; Q4, 3.75–8.51.

*^c^*SII (log) quartiles: Q1, 0.07–9.42; Q2, 9.42–10.37; Q3, 10.37–11.46; Q4, 11.46–15.51.

When the above indices were considered as categorical variables, the difference was more pronounced. SIICI stratification showed an explicit progressive correlation with 30 or 90-days mortality, the significance of which was evident from the third quartile (Q3) (30-day mortality Q3 vs. Q1: Model 1, HR [95% CI], 1.497 [1.151, 1.947], *p* = 0.003; Model 2, 1.482 [1.139, 1.929], *p* = 0.003; and Model 3, 1.415 [1.085, 1.845], *p* = 0.01; Q4 vs. Q1: Model 1, HR [95% CI], 2.430 [1.907, 3.096]; Model 2, 2.403 [1.886, 3.062]; and Model 3, 2.159 [1.688, 2.760], *p* < 0.001 in all models; 90-day mortality Q3 vs. Q1: Model 1, HR [95% CI], 1.474 [1.147, 1.894], *p* = 0.002; Model 2, 1.466 [1.140, 1.885], *p* = 0.003; and Model 3, 1.411 [1.095, 1.819], *p* = 0.008; and Q4 vs. Q1: Model 1, HR [95% CI], 2.352 [1.866, 2.964]; Model 2, 2.350 [1.864, 2.963]; and Model 3, 2.127 [1.682, 2.689], *p* < 0.001 in all models). SIRI stratification showed a stronger association with 90-day mortality than with 30-day mortality (30-day mortality Q3 vs. Q1: *p* > 0.05 in all models; Q4 vs. Q1: Model 1, HR [95% CI], 2.008 [1.589, 2.537]; Model 2, 1.915 [1.515, 2.421]; 1.689 [1.332, 2.140], *p* < 0.001 in all models; 90-day mortality Q3 vs. Q1: Model 1, HR [95% CI], 1.316 [1.036, 1.671], *p* = 0.024; Model 2, 1.304 [1.026, 1.657], *p* = 0.03; and Model 3, 1.224 [0.961, 1.558], *p* = 0.1; and Q4 vs. Q1: Model 1, HR [95% CI], 1.977 [1.581, 2.473]; Model 2, 1.909 [1.526, 2.389]; and Model 3, 1.702 [1.357, 2.136], *p* < 0.001 in all models). For the SII, only the highest quartile was associated with mortality (30-day mortality Q4 vs. Q1: Model 1, HR [95% CI], 1.604 [1.288, 1.998], *p* < 0.001; Model 2, 1.509 [1.210, 1.881], *p* < 0.001; and Model 3, 1.318 [1.052, 1.652], *p* = 0.017; 90-day mortality Q4 vs. Q1: Model 1, HR [95% CI], 1.639 [1.327, 2.025], *p* < 0.001; Model 2, 1.543 [1.248, 1.909], *p* < 0.001; and Model 3, 1.367 [1.099, 1.699], *p* = 0.005). The *p* value for trend was < 0.05 in all models (Table 3).

### 3.3 Incidence of 30 or 90-days mortality among different quartiles of the SIICI, SIRI, or SII

**FIGURE 2:**
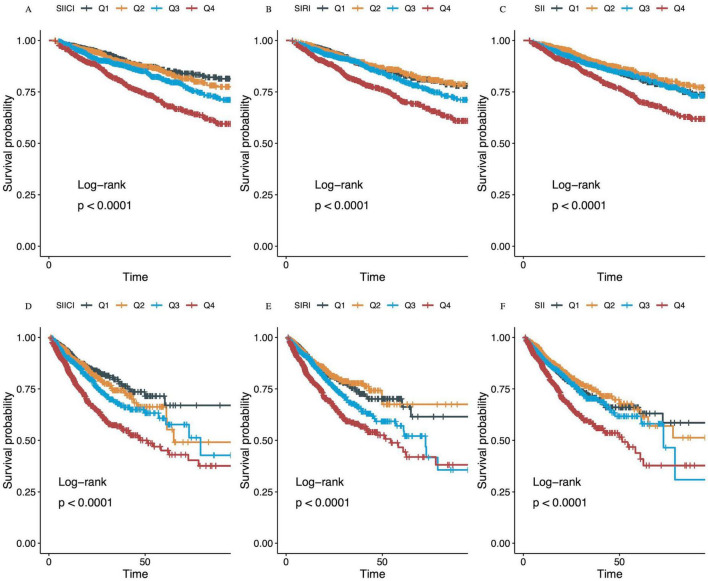
Kaplan–Meier survival analysis curves for **(A–C)** 30-day and **(D–F)** 90-day mortality. **(A,D)** SIICI (log), Q1, 0.00–3.80; Q2, 3.80–4.96; Q3, 4.96–6.19; Q4, 6.19–13.23. **(B,E)** SIRI (log), Q1, 0.00–1.69; Q2, 1.69–2.69; Q3, 2.69–3.75; Q4, 3.75–8.51. **(C,F)** SII (log), Q1, 0.07–9.42; Q2, 9.42–10.37; Q3, 10.37–11.46; Q4, 11.46–15.51. SIICI, Systemic immune-inflammatory complex index; SIRI, Systemic inflammation response index; SII, Systemic immune-inflammation index.

The strong correlation between SIICI stratification and mortality was further illustrated using Kaplan–Meier curves. Although a higher SIICI, SIRI, or SII showed a positive association with mortality at both 30 and 90 days (all *p* < 0.0001), the gradation was more pronounced in quartiles of the SIICI compared with quartiles of the SIRI or SII (Figures 2A,D). The Q1 and Q2 of the SIRI had a similar effect on mortality; the Q1 and Q3 of the SII had an almost identical effect, being higher than the Q2 in 30 and 90-days mortality (Figures 2B,C,E,F).

### 3.4 Dose–response relationship between the SIICI, SIRI, or SII and 30- or 90-days mortality

**FIGURE 3:**
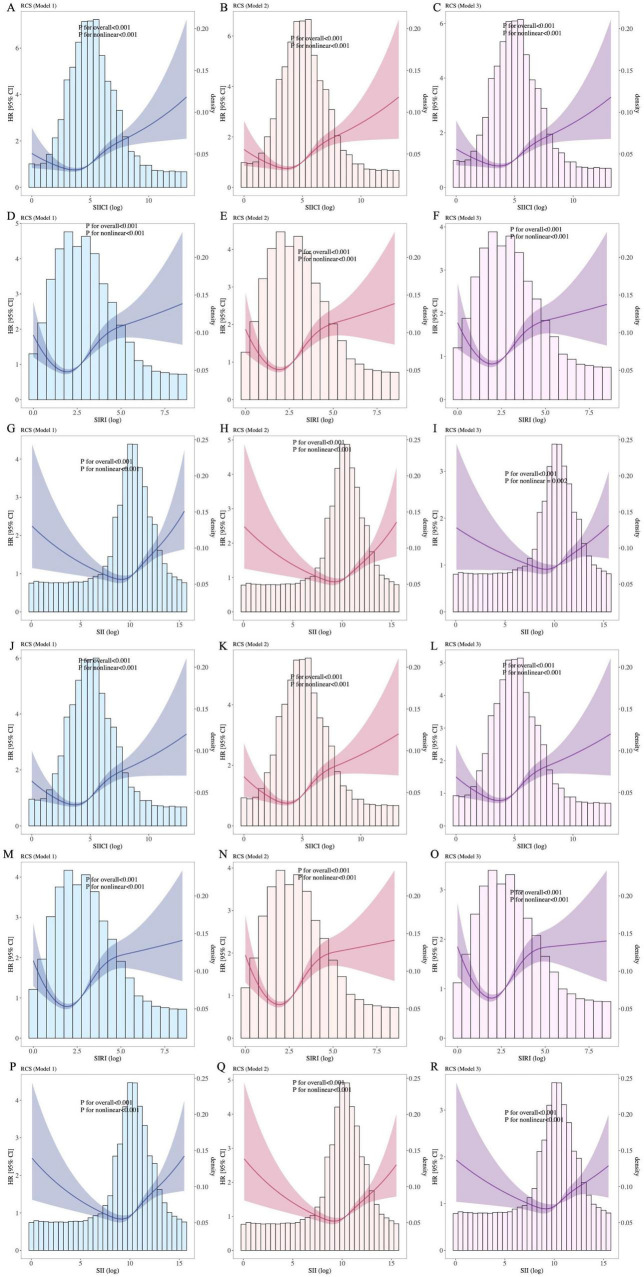
Association between indices and **(A–I)** 30-day and **(J–R)** 90-day mortality. **(A–C)** and **(J–L),** SIICI and mortality; **(D–F,M–O)**, SIRI and mortality; **(G–I)** and **(P–R)**, SII and mortality. SIICI, Systemic immune-inflammatory complex index; SIRI, Systemic inflammation response index; SII, Systemic immune-inflammation index. Model 1: Unadjusted. Model 2: Adjusted for age, sex, race and ethnicity, and marital status. Model 3: Adjusted for age, sex, race and ethnicity, marital status, heart rate, systolic blood pressure, diastolic blood pressure, mean arterial pressure, respiratory rate, hematocrit, hemoglobin, albumin, metastatic carcinoma, ischemic heart disease, chronic kidney disease, and cirrhosis.

Figure 3 depicts the non-linear relationship between the SIICI, SIRI, or SII and sepsis mortality. All *p* values for non-linearity were < 0.01 in all models. For either 30-day (Figures 3A–I) or 90-day mortality (Figures 3J–R), the SIICI and SIRI showed an S-shape, the former being milder, and the SII presented a V-shape.

### 3.5 Comparison of the prognostic value of the SIICI, SIRI, and SII for sepsis mortality

**FIGURE 4:**
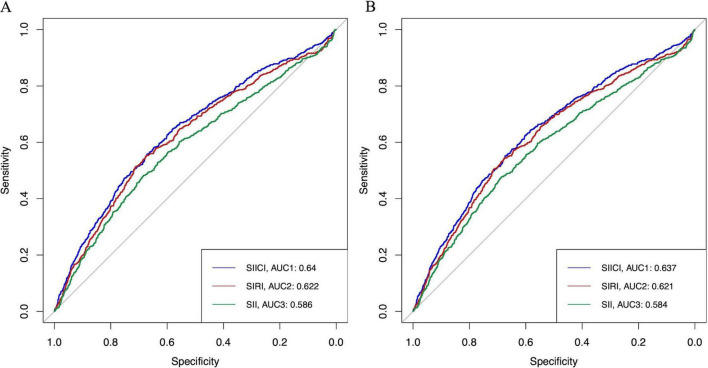
ROC of the SIICI, SIRI, and SII for **(A)** 30-day and **(B)** 90-day mortality. SIICI, Systemic immune-inflammatory complex index; SIRI, Systemic inflammation response index; SII, Systemic immune-inflammation index; ROC, Receiver operating characteristic curve; AUC, Area under the curve.

To further assess the prognostic value of the SIICI, SIRI, and SII for 30- and 90-days mortality, the ROC and AUC were plotted (Figures 4A,B). Additionally, traditional scoring systems such as the SOFA score and SAPS II were introduced to evaluate the effect of the novel indices. The AUC and Youden index of the SIICI for both 30-day and 90-day mortality were higher than those of the SIRI or the SII, but were lower than those of the SOFA score and the SAPS II. The SII had the lowest AUC and Youden index (AUCs for the SIICI, SIRI, SII, SOFA, and SAPS II for 30-day mortality were 0.64, 0.622, 0.586, 0.756, and 0.739, respectively; Youden indices for the SIICI, SIRI, SII, SOFA, and SAPS II for 30-day mortality were 0.229, 0.226, 0.166, 0.419, and 0.355, respectively; AUCs for the SIICI, SIRI, SII, SOFA, and SAPS II for 90-day mortality were 0.637, 0.621, 0.584, 0.751, and 0.745, respectively; and Youden indices for the SIICI, SIRI, SII, SOFA, and SAPS II for 90-day mortality were 0.227, 0.219, 0.164, 0.406, and 0.367, respectively). The optimal cut-off values of the SIICI (log) index for predicting 30- and 90-days mortality were 5.561 and 5.271, respectively (Table 4).

**TABLE 4:** Comparison of the prognostic value of indices and scoring systems for sepsis mortality.

Indices and scores	AUC	Optimal cut–off value	Sensitivity	Specificity	Youden index
**30–day mortality**					
SIICI (log)	0.64	5.561	0.558	0.671	0.229
SIRI (log)	0.622	3.251	0.552	0.674	0.226
SII (log)	0.586	11.014	0.483	0.683	0.166
SOFA	0.756	5.5	0.704	0.715	0.419
SAPS II	0.739	39.5	0.801	0.554	0.355
**90–day mortality**					
SIICI (log)	0.637	5.271	0.624	0.603	0.227
SIRI (log)	0.621	3.261	0.54	0.679	0.219
SII (log)	0.584	11.035	0.474	0.69	0.164
SOFA	0.751	5.5	0.688	0.718	0.406
SAPS II	0.745	41.5	0.732	0.635	0.367

AUC, Area under the curve of receiver operating characteristic; SOFA, Sequential organ failure assessment; SAPS II, Simplified acute physiology score II.

### 3.6 Association between the SIICI and secondary endpoints

The association between the SIICI and secondary outcomes was conducted based on PSM, in which patients were matched and divided into two groups with the median SIICI (log). The results showed that both ICU and hospital stays were shorter in the group with a lower SIICI (*p* < 0.001 and *p* = 0.022, respectively) than in the group with a higher SIICI; the incidence of AKI and the use of RRT and ventilation were increased with a higher SIICI (all *p* < 0.001; Table 5).

**TABLE 5:** Association of the SIICI and secondary outcomes.

Secondary outcomes	SIICI (log) ≤ 4.96, *n* = 1,520^1^	SIICI (log) > 4.96, *n* = 1,520^1^	*p*-value
ICU stay, day	3 [2, 8]	4 [2, 9]	<0.001
Hospital stay, day	11 [6, 20]	12 [7, 21]	0.022
AKI	630 (41%)	772 (51%)	<0.001
RRT	147 (9.7%)	237 (16%)	<0.001
Ventilation	828 (54%)	934 (61%)	<0.001

AKI, Acute kidney injury; RRT, Renal replacement therapy. Continuous variables are described as the median and interquartile range (IQR) (median [IQR]), categorical variables are described as frequencies and percentages [n (%)].

### 3.7 Subgroup analysis

**FIGURE 5:**
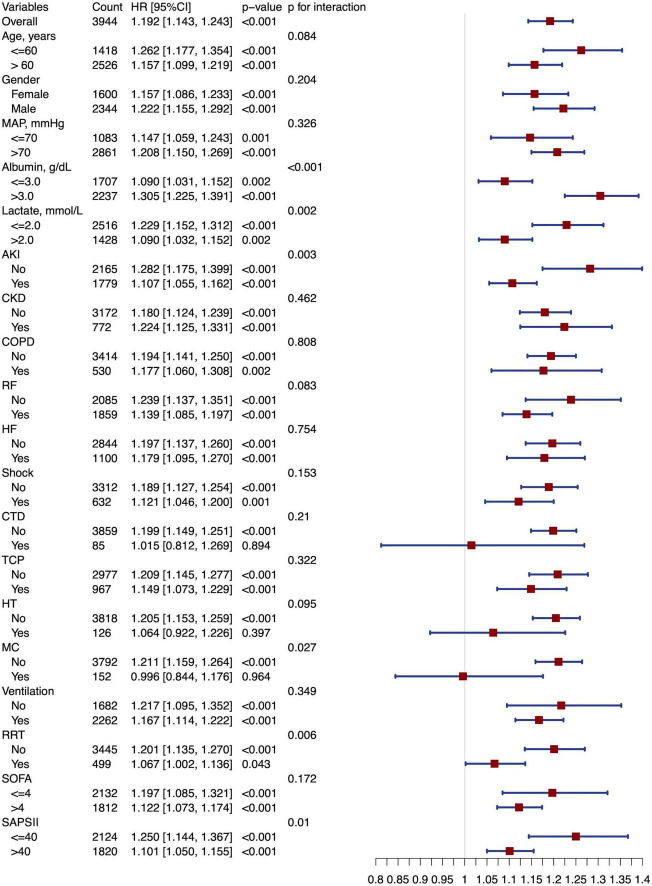
Forest plot of subgroup analysis. MAP, Mean arterial pressure; AKI, Acute kidney injury; CKD, Chronic kidney disease; COPD, Chronic obstructive pulmonary disease; RF, Respiratory failure; HF, Heart failure; CTD, Connective tissue disease; TCP, Thrombocytopenia; HT, Hematological tumors; MC, Metastatic carcinoma; RRT, Renal replacement therapy; SOFA, Sequential organ failure assessment; SAPS II, Simplified acute physiology score II.

Subgroup analysis showed that a higher SIICI was associated with increased mortality in subgroups with an absence of CTD (HR [95% CI]: 1.199 [1.149, 1.251], *p* < 0.001) or MC (HR [95% CI]: 1.211 [1.159, 1.264], *p* < 0.001). Evident interactions were observed in subgroups of albumin (*p* < 0.001), lactate (*p* = 0.002), AKI (*p* = 0.003), MC (*p* = 0.027), RRT (*p* = 0.006), and SAPS II (*p* = 0.01) (Figure 5).

## 4 Discussion

In this study, we first proposed and demonstrated that the SIICI, which is calculated as (neutrophil × monocyte count) × 10^3^/(platelet × lymphocyte count), was positively associated with higher 30- and 90-days mortality and had a better prognostic value than the SIRI and SII.

Mangalesh et al. and Li et al. had previously integrated the same four components in the systemic immune-inflammatory response index (SIIRI), but this index was defined as platelet × monocyte × neutrophil count/lymphocyte count, and it was used to predict the outcome of coronary artery disease (CAD) (26, 27). Although the underlying pathophysiological manifestation of both CAD and sepsis is inflammation, the mechanisms are distinct and lead to different types of harm. In CAD, platelets are involved not only in the pathogenesis of atherosclerosis, but also in the development of acute thrombotic events through activation, adhesion, and aggregation to form coronary thrombosis (28, 29). Accordingly, a reduction in the platelet count or activity is not usually observed. A similar situation can be extended to solid tumor development: platelets play a critical role by secreting microparticles containing multiple proliferative factors, reducing leukocyte apoptosis, inducing angiogenesis, supporting tumor stem cells, and promoting metastasis, and thrombocytopenia does not commonly occur in non-hematological tumors (28). Additionally, previous studies have illustrated that an increase in leukocytes is associated with tumor growth; thus, the PLR and NLR have reasonably become promising prognostic indicators of solid tumor prognosis (30–34).

In contrast to this, the incidence of thrombocytopenia in patients with sepsis is approximately 30–60% and is positively correlated with mortality and the severity of illness (13, 14). In patients with sepsis, thrombocytopenia has several causes, including increased consumption or destruction of platelets (e.g., disseminated intravascular coagulation, ADAMTS13 depletion, or hemophagocytic syndrome), decreased platelet production owing to myelosuppression and the phagocytosis of monocytes and macrophages, or excessive platelet loss due to surgery or mechanical organ support devices (11, 35–37). Unsurprisingly, thrombocytopenia has been shown to be positively associated with poor prognosis in sepsis (10–12). Lymphocytopenia is also strongly associated with mortality (7–9) and may occur in the early stages of sepsis and persist over time. Both neutrophils and monocytes are involved in the early immune response and increase in neutrophils in the early stages of sepsis (38). However, an increase in neutrophils is not necessarily associated with a poor prognosis. Although some studies have shown that an increase in the neutrophil count may be associated with the intensity of the inflammatory response, its relationship to prognosis is complex (39). As the inflammatory response progresses, monocytes differentiate into macrophages and continue to participate in it (39). Based on the above, the SIICI index has been proposed to magnify the inflammatory status and may be used to stratify patients: in mild cases, without significant reductions in lymphocytes and platelets, SIICI indicators may be normal or slightly elevated; in contrast, critically ill patients may exhibit a particularly significant increase in SIICI due to the decrease in neutrophils and monocytes caused by immunosuppression, along with a significant decrease in lymphocytes and platelets.

Compared to SIICI, SIRI does not include the platelet count, and thus omits a critical factor involved in sepsis. The SII not only omits the monocyte count, but also places the platelet and neutrophil counts together in the numerator position, while the lymphocyte count serves as the denominator. In critically ill patients with both lymphopenia and thrombocytopenia, this may cause the results to be offset and as such may not accurately reflect the severity of the condition.

Consistent with the above theory, our study showed that the SIICI comprehensively outperformed both the SII and SIRI. A Cox proportional hazards model and Kaplan–Meier curve showed that the risks of both 30- and 90-days mortality were well distributed in all models according to different stratifications of the SIICI, whereas the SII performed the poorest. Similarly, the SIICI had the highest value of both the AUC and Youden index among the three novel indices, indicating a more promising predictive potential, albeit lower than that of traditional scoring systems such as the SOFA score and SAPS II. When it comes to the linear relationship between these indices and mortality, the SIICI was closest to linearity, as compared with the S-shape of the SIRI and the V-shape of the SII. Moreover, the V-shaped relationship between the SII and outcome implies an SII that is too low (neutropenia or thrombocytopenia predominant, or both), as well as one that is too high (lymphocytopenia predominant) is correlated with greater mortality. The offset of two variables in the same direction of change inevitably leads to a decrease in sensitivity, which is directly evidenced by our study findings that the SII had the lowest sensitivity among all indices. Taken together, we can conclude that the SIICI is a more promising predictor of sepsis mortality than the SII and SIRI.

Regarding secondary outcomes, both ICU and hospital stays were 1 day longer in the group with a higher SIICI than in the group with a lower SIICI. Additionally, the incidence of AKI and the use of RRT or mechanical ventilation were significantly higher in the higher SIICI group than in the lower SIICI group, further indicating that the SIICI has an ideal prognostic effect.

Subgroup analysis was performed to evaluate potential confounders of the SIICI. The results showed a negative association between the SIICI level and the presence of CTD, HT, and MC, all of which are likely to cause alteration in the leukocyte or platelet count. Additionally, there was an interaction in subgroups with or without MC. The underlying reason may be because the change is mainly induced by primary diseases rather than acute inflammation, and other factors such as treatment selection or genotyping dominate the association with the outcome. However, these results should be interpreted with caution because the sample in these subgroups was small.

In this study, we first proposed the SIICI to predict a poor prognosis of sepsis, which performed better than the SIRI or SII. Although the prognostic value was lower than that of the SOFA score or SAPS II, the SIICI is based on a routine blood test that is easily and inexpensively obtained in clinical practice. The SIICI can serve as a preliminary screening tool to quickly identify high-risk patients who may require closer monitoring and management in resource-limited clinical settings. The SIICI may also serve as an adjunct risk stratification tool to help clinicians identify high-risk patients and adjust treatment plans in a timely manner.

This study has several limitations. First, this was a single-center retrospective study; therefore, selection bias could not be avoided. Second, we did not investigate the change trend in the SIICI during the study period; therefore, the impact of dynamic change on the prognosis of sepsis cannot be determined. Third, confounders such as HT and MC are commonly found in patients with sepsis; a larger sample is required to evaluate the interaction with inflammation. Finally, evidence is lacking regarding mechanisms illustrating the association between the SIICI and sepsis mortality.

## 5 Conclusion

We first proposed the SIICI as a novel indicator to predict sepsis prognosis. Our study showed that the SIICI was strongly associated with 30 and 90-days mortality and that the prognostic value was better than those of the SIRI and SII. Additionally, a higher SIICI was related to a longer ICU or hospital stay, higher incidence of AKI, and greater use of RRT and mechanical ventilation. Although the SIICI did not reach the predictive level of the SOFA score and SAPS II, it is easy to obtain and inexpensive, and thus may serve as a preliminary screening and risk stratification tool. The SIICI is easier to implement, especially in resource-limited settings, and may help clinicians identify high-risk patients and adjust treatment plans in a timely manner.

## Data Availability

The datasets presented in this study can be found in online repositories. The names of the repository/repositories and accession number(s) can be found in the article/Supplementary material.
