# Supplementary material for: Systemic immune-inflammatory complex index as a novel predictor of sepsis prognosis: a retrospective cohort study using MIMIC-IV
Source: Front Med (Lausanne). 2025 Jun 30;12:1608619. doi: 10.3389/fmed.2025.1608619 (PMC12258050; doi:10.3389/fmed.2025.1608619)
Supplement: Supplementary file 1 [file Table_1.docx]

**Supplementary materials:**

**Table S1. Baseline characteristics of sepsis patients according to the quartiles of the SIRI (log).**

| **Characteristics** | **Overall,**  **n = 3,944*^1^*** | **Q1,**  **n = 986*^1^*** | **Q2,**  **n = 986*^1^*** | **Q3,**  **n = 986*^1^*** | **Q4,**  **n = 986*^1^*** | ***p*–value*^2^*** |
| --- | --- | --- | --- | --- | --- | --- |
| 30–day mortality | 609 (15%) | 99 (10%) | 108 (11%) | 157 (16%) | 245 (25%) | <0.001*** |
| 90–day mortality | 663 (17%) | 109 (11%) | 113 (11%) | 178 (18%) | 263 (27%) | <0.001*** |
| In–hospital mortality | 665 (17%) | 109 (11%) | 113 (11%) | 180 (18%) | 263 (27%) | <0.001*** |
| ICU stay, day | 4 [2,8] | 3 [1,6] | 4 [2,8] | 5 [2,9] | 5 [2,9] | <0.001*** |
| Hospital stay, day | 11 [6,21] | 8 [6,17] | 11 [6,19] | 14 [7,22] | 13 [7,23] | <0.001*** |
| Marital status |  |  |  |  |  | <0.001*** |
| Married | 1,588 (40%) | 452 (46%) | 419 (42%) | 360 (37%) | 357 (36%) |  |
| Not married | 2,356 (60%) | 534 (54%) | 567 (58%) | 626 (63%) | 629 (64%) |  |
| Race and ethnicity |  |  |  |  |  | 0.022* |
| Nonwhite | 1,621 (41%) | 393 (40%) | 397 (40%) | 385 (39%) | 446 (45%) |  |
| White | 2,323 (59%) | 593 (60%) | 589 (60%) | 601 (61%) | 540 (55%) |  |
| Gender |  |  |  |  |  | 0.4 |
| Female | 1,600 (41%) | 418 (42%) | 381 (39%) | 394 (40%) | 407 (41%) |  |
| Male | 2,344 (59%) | 568 (58%) | 605 (61%) | 592 (60%) | 579 (59%) |  |
| Age, years | 66 [55,76] | 66 [55,75] | 66 [55,76] | 67 [55,77] | 66 [55,78] | 0.2 |
| **Vital Signs** |  |  |  |  |  |  |
| Heart rate, beats/min | 87 [77,102] | 81 [74,96] | 86 [76,100] | 90 [79,105] | 93 [79,108] | <0.001*** |
| Systolic blood pressure, mmHg | 117 [103,134] | 116 [101,130] | 116 [102,132] | 118 [104,137] | 120 [105,137] | <0.001*** |
| Diastolic blood pressure, mmHg | 67 [57,79] | 65 [55,77] | 67 [57,79] | 68 [58,81] | 68 [58,81] | <0.001*** |
| MAP, mmHg | 80 [69,93] | 77 [68,90] | 79 [69,92] | 81 [71,93] | 82 [71,95] | <0.001*** |
| Respiratory rate, times/min | 19 [15,23] | 17 [14,22] | 18 [15,22] | 19 [16,24] | 20 [17,25] | <0.001*** |
| SaO_2_, % | 98.0 [95.0,100.0] | 99.0 [96.0,100.0] | 98.0 [96.0,100.0] | 98.0 [95.0,100.0] | 97.0 [94.0,100.0] | <0.001*** |
| **Laboratory indicators** |  |  |  |  |  |  |
| SIRI | 5 [2,12] | 1 [1,2] | 3 [3,4] | 8 [7,10] | 23 [16,35] | <0.001*** |
| SIRI (log) | 2.69 [1.69,3.75] | 1.11 [0.72,1.42] | 2.17 [1.93,2.44] | 3.19 [2.94,3.46] | 4.57 [4.12,5.18] | <0.001*** |
| Lymphocyte count, 10^9^/L | 1.21 [0.73,1.85] | 1.57 [0.92,2.36] | 1.45 [0.95,2.09] | 1.17 [0.77,1.64] | 0.80 [0.47,1.21] | <0.001*** |
| Monocyte count, 10^9^/L | 0.71 [0.42,1.08] | 0.32 [0.18,0.49] | 0.63 [0.45,0.84] | 0.90 [0.65,1.14] | 1.21 [0.85,1.73] | <0.001*** |
| Neutrocyte count, 10^9^/L | 10 [6,14] | 5 [3,8] | 8 [6,11] | 11 [8,14] | 16 [12,21] | <0.001*** |
| Platelet count, 10^9^/L | 181 [129,238] | 158 [108,208] | 179 [131,233] | 194 [139,248] | 195 [138,261] | <0.001*** |
| White blood cell count, 10^9^/L | 11 [8,16] | 7 [5,10] | 10 [8,13] | 13 [10,16] | 17 [13,22] | <0.001*** |
| Hematocrit, % | 34 [29,39] | 34 [28,39] | 34 [29,39] | 34 [29,39] | 33 [29,39] | 0.064 |
| Hemoglobin, g/dL | 11.00 [9.30,12.80] | 11.00 [9.10,12.70] | 11.30 [9.33,12.90] | 11.00 [9.30,12.80] | 10.80 [9.20,12.60] | 0.072 |
| Albumin, g/dL | 3.10 [2.70,3.57] | 3.30 [2.90,3.80] | 3.20 [2.80,3.60] | 3.10 [2.70,3.50] | 2.99 [2.60,3.30] | <0.001*** |
| Serum creatinine, mg/dL | 1.00 [0.80,1.50] | 0.90 [0.70,1.20] | 1.00 [0.70,1.30] | 1.10 [0.80,1.50] | 1.20 [0.80,1.98] | <0.001*** |
| Urea nitrogen, mg/dL | 19 [14,31] | 17 [13,25] | 18 [13,26] | 20 [14,34] | 24 [16,41] | <0.001*** |
| Total bilirubin, mg/dL | 0.70 [0.40,1.30] | 0.70 [0.40,1.10] | 0.66 [0.40,1.09] | 0.70 [0.40,1.40] | 0.80 [0.50,1.70] | <0.001*** |
| Glucose, mg/dL | 125 [102,162] | 117 [98,148] | 123 [102,153] | 127 [104,164] | 136 [108,180] | <0.001*** |
| INR | 1.30 [1.10,1.50] | 1.20 [1.10,1.40] | 1.20 [1.10,1.50] | 1.20 [1.10,1.50] | 1.30 [1.10,1.60] | <0.001*** |
| APTT, s | 31 [27,37] | 31 [28,37] | 30 [27,37] | 30 [27,36] | 31 [27,38] | 0.010* |
| Sodium, mmol/L | 139.0 [136.0,142.0] | 139.0 [137.0,142.0] | 139.0 [136.0,142.0] | 139.0 [136.0,142.0] | 138.0 [135.0,141.0] | <0.001*** |
| Potassium, mmol/L | 4.10 [3.70,4.60] | 4.10 [3.73,4.40] | 4.10 [3.73,4.50] | 4.10 [3.80,4.60] | 4.20 [3.70,4.70] | <0.001*** |
| Bicarbonate, mmol/L | 22.0 [20.0,25.0] | 23.0 [21.0,25.0] | 23.0 [20.0,25.0] | 22.0 [19.0,25.0] | 21.0 [18.0,24.0] | <0.001*** |
| Lactate, mmol/L | 1.70 [1.20,2.50] | 1.60 [1.11,2.20] | 1.60 [1.20,2.20] | 1.76 [1.30,2.60] | 2.00 [1.40,3.10] | <0.001*** |
| PO_2_, mmHg | 104 [54,224] | 181 [79,359] | 118 [62,275] | 91 [50,165] | 77 [46,128] | <0.001*** |
| **Comorbidities** |  |  |  |  |  |  |
| AKI | 1,779 (45%) | 327 (33%) | 379 (38%) | 490 (50%) | 583 (59%) | <0.001*** |
| CKD | 772 (20%) | 151 (15%) | 180 (18%) | 198 (20%) | 243 (25%) | <0.001*** |
| COPD | 530 (13%) | 96 (9.7%) | 120 (12%) | 143 (15%) | 171 (17%) | <0.001*** |
| Respiratory failure, RF | 1,859 (47%) | 315 (32%) | 412 (42%) | 529 (54%) | 603 (61%) | <0.001*** |
| Ischemic heart disease, IHD | 680 (17%) | 151 (15%) | 172 (17%) | 167 (17%) | 190 (19%) | 0.14 |
| Heart failure, HF | 1,100 (28%) | 219 (22%) | 250 (25%) | 296 (30%) | 335 (34%) | <0.001*** |
| Shock | 632 (16%) | 96 (9.7%) | 133 (13%) | 166 (17%) | 237 (24%) | <0.001*** |
| Connective tissue disease, CTD | 85 (2.2%) | 22 (2.2%) | 14 (1.4%) | 22 (2.2%) | 27 (2.7%) | 0.2 |
| Cirrhosis | 9 (0.2%) | 1 (0.1%) | 3 (0.3%) | 3 (0.3%) | 2 (0.2%) | 0.9 |
| Thrombocytopenia, TCP | 967 (25%) | 264 (27%) | 228 (23%) | 231 (23%) | 244 (25%) | 0.2 |
| Hematologic tumor, HT | 126 (3.2%) | 61 (6.2%) | 16 (1.6%) | 16 (1.6%) | 33 (3.3%) | <0.001*** |
| Metastatic carcinoma, MC | 152 (3.9%) | 31 (3.1%) | 33 (3.3%) | 37 (3.8%) | 51 (5.2%) | 0.083 |
| AIDS | 17 (0.4%) | 13 (1.3%) | 3 (0.3%) | 1 (0.1%) | 0 (0%) | <0.001*** |
| **Operations** |  |  |  |  |  |  |
| Ventilation | 2,262 (57%) | 479 (49%) | 555 (56%) | 595 (60%) | 633 (64%) | <0.001*** |
| RRT | 499 (13%) | 96 (9.7%) | 85 (8.6%) | 120 (12%) | 198 (20%) | <0.001*** |
| **Scoring systems** |  |  |  |  |  |  |
| SOFA | 4 [2,7] | 4 [3,6] | 4 [2,6] | 4 [2,7] | 5 [3,8] | <0.001*** |
| SAPS II | 40 [33,47] | 36 [31,43] | 38 [32,45] | 41 [34,48] | 43 [37,51] | <0.001*** |

SIRI (log) quartiles: Q1, 0.00–1.69; Q2, 1.69–2.69; Q3, 2.69–3.75; Q4, 3.75–8.51.

MAP, mean arterial pressure; SaO_2_, oxygen saturation; INR, international normalized ratio; APTT, activated partial thromboplastin time; PO_2_, partial pressure of oxygen; AKI, acute kidney injury; CKD, chronic kidney disease; COPD, chronic obstructive pulmonary disease; AIDS, acquired immunodeficiency syndrome; RRT, renal replacement therapy; SOFA, sequential organ failure assessment; SAPS II, simplified acute physiology score II.

*^1^*Continuous variables were described as median and interquartile range (IQR) (median [IQR]), categorical variables were described as frequencies and percentages (n (%)).

*^2^* **p* <0.05; ***p* <0.01; ****p* <0.001.

**Table S2. Baseline characteristics of sepsis patients according to the quartiles of the SII (log).**

| **Characteristics** | **Overall,**  **n = 3,944*^1^*** | **Q1,**  **n = 986*^1^*** | **Q2,**  **n = 986*^1^*** | **Q3,**  **n = 986*^1^*** | **Q4,**  **n = 986*^1^*** | ***p*–value*^2^*** |
| --- | --- | --- | --- | --- | --- | --- |
| 30–day mortality | 609 (15%) | 124 (13%) | 109 (11%) | 152 (15%) | 224 (23%) | <0.001*** |
| 90–day mortality | 663 (17%) | 134 (14%) | 121 (12%) | 167 (17%) | 241 (24%) | <0.001*** |
| In–hospital mortality | 665 (17%) | 134 (14%) | 123 (12%) | 167 (17%) | 241 (24%) | <0.001*** |
| ICU stay, day | 4 [2,8] | 3 [1,7] | 3 [2,8] | 5 [2,10] | 4 [2,9] | <0.001*** |
| Hospital stay, day | 11 [6,21] | 9 [6,19] | 10 [6,20] | 13 [7,23] | 12 [7,21] | <0.001*** |
| Marital status |  |  |  |  |  | 0.002** |
| Married | 1,588 (40%) | 413 (42%) | 435 (44%) | 382 (39%) | 358 (36%) |  |
| Not married | 2,356 (60%) | 573 (58%) | 551 (56%) | 604 (61%) | 628 (64%) |  |
| Race and ethnicity |  |  |  |  |  | 0.010* |
| Nonwhite | 1,621 (41%) | 401 (41%) | 370 (38%) | 407 (41%) | 443 (45%) |  |
| White | 2,323 (59%) | 585 (59%) | 616 (62%) | 579 (59%) | 543 (55%) |  |
| Gender |  |  |  |  |  | <0.001*** |
| Female | 1,600 (41%) | 390 (40%) | 338 (34%) | 407 (41%) | 465 (47%) |  |
| Male | 2,344 (59%) | 596 (60%) | 648 (66%) | 579 (59%) | 521 (53%) |  |
| Age, years | 66 [55,76] | 64 [54,74] | 66 [54,76] | 66 [55,76] | 67 [56,78] | <0.001*** |
| **Vital Signs** |  |  |  |  |  |  |
| Heart rate, beats/min | 87 [77,102] | 84 [75,99] | 84 [75,99] | 88 [77,103] | 93 [80,107] | <0.001*** |
| Systolic blood pressure, mmHg | 117 [103,134] | 116 [101,132] | 116 [102,131] | 117 [104,136] | 121 [105,137] | 0.001** |
| Diastolic blood pressure, mmHg | 67 [57,79] | 66 [56,77] | 67 [56,77] | 68 [58,82] | 68 [58,80] | 0.002** |
| MAP, mmHg | 80 [69,93] | 78 [69,92] | 79 [69,91] | 81 [70,94] | 81 [71,94] | <0.001*** |
| Respiratory rate, times/min | 19 [15,23] | 18 [15,23] | 18 [15,22] | 19 [16,23] | 20 [17,25] | <0.001*** |
| SaO_2_, % | 98.0 [95.0,100.0] | 99.0 [96.0,100.0] | 99.0 [96.0,100.0] | 98.0 [95.0,100.0] | 97.0 [94.0,100.0] | <0.001*** |
| **Laboratory indicators** |  |  |  |  |  |  |
| SII | 1,324 [682,2,814] | 398 [255,538] | 975 [822,1,131] | 1,869 [1,553,2,274] | 4,981 [3,548,7,639] | <0.001*** |
| SII (log) | 10.37 [9.42,11.46] | 8.64 [8.00,9.07] | 9.93 [9.69,10.14] | 10.87 [10.60,11.15] | 12.28 [11.79,12.90] | <0.001*** |
| Lymphocyte count, 10^9^/L | 1.21 [0.73,1.85] | 1.75 [1.05,2.58] | 1.48 [1.02,2.07] | 1.18 [0.81,1.59] | 0.68 [0.40,1.00] | <0.001*** |
| Monocyte count, 10^9^/L | 0.71 [0.42,1.08] | 0.52 [0.29,0.81] | 0.68 [0.42,1.00] | 0.86 [0.53,1.19] | 0.84 [0.49,1.26] | <0.001*** |
| Neutrocyte count, 10^9^/L | 10 [6,14] | 6 [3,8] | 9 [6,12] | 11 [8,14] | 15 [11,20] | <0.001*** |
| Platelet count, 10^9^/L | 181 [129,238] | 125 [80,167] | 169 [127,215] | 204 [158,251] | 234 [176,314] | <0.001*** |
| White blood cell count, 10^9^/L | 11 [8,16] | 8 [6,11] | 10 [8,14] | 12 [9,16] | 16 [12,22] | <0.001*** |
| Hematocrit, % | 34 [29,39] | 32 [27,38] | 35 [29,40] | 35 [30,40] | 33 [29,39] | <0.001*** |
| Hemoglobin, g/dL | 11.00 [9.30,12.80] | 10.50 [8.70,12.30] | 11.50 [9.50,13.10] | 11.40 [9.60,13.08] | 10.70 [9.20,12.50] | <0.001*** |
| Albumin, g/dL | 3.10 [2.70,3.57] | 3.20 [2.80,3.60] | 3.22 [2.89,3.70] | 3.17 [2.70,3.58] | 2.92 [2.60,3.30] | <0.001*** |
| Serum creatinine, mg/dL | 1.00 [0.80,1.50] | 0.90 [0.70,1.30] | 1.00 [0.80,1.30] | 1.00 [0.80,1.50] | 1.20 [0.80,1.80] | <0.001*** |
| Urea nitrogen, mg/dL | 19 [14,31] | 18 [13,27] | 18 [14,26] | 20 [14,31] | 24 [16,39] | <0.001*** |
| Total bilirubin, mg/dL | 0.70 [0.40,1.30] | 0.80 [0.50,1.50] | 0.70 [0.40,1.11] | 0.70 [0.40,1.19] | 0.70 [0.40,1.30] | <0.001*** |
| Glucose, mg/dL | 125 [102,162] | 117 [98,145] | 123 [101,153] | 127 [104,167] | 137 [109,181] | <0.001*** |
| INR | 1.30 [1.10,1.50] | 1.30 [1.10,1.50] | 1.20 [1.10,1.40] | 1.20 [1.10,1.50] | 1.30 [1.10,1.50] | <0.001*** |
| APTT, s | 31 [27,37] | 31 [28,38] | 31 [27,36] | 30 [27,37] | 30 [27,36] | 0.007** |
| Sodium, mmol/L | 139.0 [136.0,142.0] | 139.0 [136.0,142.0] | 139.0 [136.0,141.0] | 139.0 [136.0,142.0] | 138.0 [135.0,141.0] | <0.001*** |
| Potassium, mmol/L | 4.10 [3.70,4.60] | 4.10 [3.70,4.50] | 4.10 [3.70,4.50] | 4.10 [3.80,4.60] | 4.20 [3.80,4.70] | <0.001*** |
| Bicarbonate, mmol/L | 22.0 [20.0,25.0] | 23.0 [20.0,25.0] | 23.0 [20.0,25.0] | 22.0 [20.0,25.0] | 21.0 [18.0,24.0] | <0.001*** |
| Lactate, mmol/L | 1.70 [1.20,2.50] | 1.60 [1.20,2.40] | 1.60 [1.20,2.20] | 1.70 [1.20,2.50] | 1.90 [1.40,3.00] | <0.001*** |
| PO_2_, mmHg | 104 [54,224] | 138 [65,317] | 135 [63,305] | 96 [54,195] | 78 [46,127] | <0.001*** |
| **Comorbidities** |  |  |  |  |  |  |
| AKI | 1,779 (45%) | 374 (38%) | 378 (38%) | 471 (48%) | 556 (56%) | <0.001*** |
| CKD | 772 (20%) | 169 (17%) | 163 (17%) | 207 (21%) | 233 (24%) | <0.001*** |
| COPD | 530 (13%) | 96 (9.7%) | 121 (12%) | 132 (13%) | 181 (18%) | <0.001*** |
| Respiratory failure, RF | 1,859 (47%) | 343 (35%) | 420 (43%) | 521 (53%) | 575 (58%) | <0.001*** |
| Ischemic heart disease, IHD | 680 (17%) | 137 (14%) | 171 (17%) | 197 (20%) | 175 (18%) | 0.004** |
| Heart failure, HF | 1,100 (28%) | 212 (22%) | 244 (25%) | 324 (33%) | 320 (32%) | <0.001*** |
| Shock | 632 (16%) | 114 (12%) | 129 (13%) | 175 (18%) | 214 (22%) | <0.001*** |
| MODS | 48 (1.2%) | 15 (1.5%) | 9 (0.9%) | 9 (0.9%) | 15 (1.5%) | 0.4 |
| Connective tissue disease, CTD | 85 (2.2%) | 18 (1.8%) | 19 (1.9%) | 16 (1.6%) | 32 (3.2%) | 0.054 |
| Cirrhosis | 9 (0.2%) | 3 (0.3%) | 1 (0.1%) | 3 (0.3%) | 2 (0.2%) | 0.9 |
| Thrombocytopenia, TCP | 967 (25%) | 333 (34%) | 281 (28%) | 182 (18%) | 171 (17%) | <0.001*** |
| Hematologic tumor, HT | 126 (3.2%) | 72 (7.3%) | 14 (1.4%) | 14 (1.4%) | 26 (2.6%) | <0.001*** |
| Metastatic carcinoma, MC | 152 (3.9%) | 28 (2.8%) | 25 (2.5%) | 33 (3.3%) | 66 (6.7%) | <0.001*** |
| AIDS | 17 (0.4%) | 12 (1.2%) | 2 (0.2%) | 1 (0.1%) | 2 (0.2%) | <0.001*** |
| **Operations** |  |  |  |  |  |  |
| Ventilation | 2,262 (57%) | 506 (51%) | 562 (57%) | 601 (61%) | 593 (60%) | <0.001*** |
| RRT | 499 (13%) | 117 (12%) | 105 (11%) | 120 (12%) | 157 (16%) | 0.003** |
| **Scoring systems** |  |  |  |  |  |  |
| SOFA | 4 [2,7] | 5 [3,7] | 4 [2,6] | 4 [2,6] | 4 [2,7] | <0.001*** |
| SAPS II | 40 [33,47] | 38 [31,45] | 38 [32,45] | 40 [33,46] | 43 [37,50] | <0.001*** |

SII (log) quartiles: Q1, 0.07–9.42; Q2, 9.42–10.37; Q3, 10.37–11.46; Q4, 11.46–15.51.

MAP, mean arterial pressure; SaO_2_, oxygen saturation; INR, international normalized ratio; APTT, activated partial thromboplastin time; PO_2_, partial pressure of oxygen; AKI, acute kidney injury; CKD, chronic kidney disease; COPD, chronic obstructive pulmonary disease; AIDS, acquired immunodeficiency syndrome; RRT, renal replacement therapy; SOFA, sequential organ failure assessment; SAPS II, simplified acute physiology score II.

*^1^*Continuous variables were described as median and interquartile range (IQR) (median [IQR]), categorical variables were described as frequencies and percentages (n (%)).

*^2^* **p* <0.05; ***p* <0.01; ****p* <0.001.
